# Pyrite‐assisted denitrification in recirculated biofilter tolerates pH lower than 5

**DOI:** 10.1002/wer.10721

**Published:** 2022-05-02

**Authors:** Francesco Di Capua, Giovanni Esposito

**Affiliations:** ^1^ Department of Civil, Environmental, Land, Building Engineering and Chemistry Polytechnic University of Bari Bari Italy; ^2^ Department of Civil, Architectural and Environmental Engineering University of Naples Federico II Naples Italy

**Keywords:** autotrophic denitrification, biofilter, mining water, nitrogen removal, pyrite6

## Abstract

This study demonstrates the feasibility of pyrite‐assisted denitrification to treat mildly acidic wastewaters and proposes a convenient solution for nitrogen removal from acidic mining waters. Pyrite is a highly available and low‐cost electron donor for autotrophic denitrification, whereas mining waters represent a source of nitrogen (N) contamination due to the use of N‐containing explosives during mining operations and cyanide (CN)‐based compounds for ore processing. In this study, the denitrification performance of a recirculated pyrite‐packed biofilter (RPPB) treating simulated N‐contaminated acidic mining waters is evaluated under decreasing pH conditions. The effect of feed organic carbon on the autotrophic process has been also investigated. N removal efficiencies (NRE) > 60% could be achieved at feed pH ≥ 4.5 and HRT ≥ 5 h. Decrease of influent pH to 3.0 reduced the NRE to 20%. The addition of 10 and 20 mg/L of ethanol to the influent did not significantly impact NRE of the RPPB.

**Practitioner Points:**

- Pyrite‐assisted denitrification is proposed for treating acidic wastewaters.
- Nitrogen removal >60% was maintained at feed pH as low as 4.7.
- Decrease of feed pH to 3 strongly inhibited denitrification.
- The presence of organic carbon in the feed did not affect the autotrophic process.

## Graphical abstract

Effect of feed pH decrease on N removal via pyrite‐driven denitrification in a recirculated biofilter.

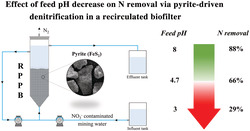

## INTRODUCTION

Mine drainage is typically generated when rain and/or sub‐surface water enter the mine or encounter waste rock dumps, tailing ponds or piles, or coal spoils, being commonly displaced in the surroundings of the mining area (Bigham & Cravotta, 2016). Rock exposure to water and air can generate acidity due to the aerobic oxidation of metal sulfides such as pyrite (FeS_2_) (Equation 1), leading to the production of acidic mine drainage (AMD) with a pH that typically ranges between 0.5 and 6.5 (Bigham & Cravotta, 2016).

(1) $$4\;{\mathrm{FeS}}_{2\;\left(s\right)}+15\;O_{2\;\left(\mathrm{aq}\right)}+14\;H_{2}O_{\left(\mathrm{aq}\right)}\to 4\;\mathrm{Fe}{\left(\mathrm{OH}\right)}_{3\;\left(\mathrm{aq}\right)}+8\;SO_{4\;\left(\mathrm{aq}\right)}^{2-}+16\;H_{\left(\mathrm{aq}\right)}^{+}$$

Besides the low pH, mining effluents may also contain high levels of nitrate (NO_3_^−^), which mainly originate from the dissolution of undetonated explosives applied for blasting operations and may cause eutrophication of the receiving waters (Nilsson & Widerlund, 2017).

Removal of NO_3_^−^ from wastewater can be carried out through chemical or biological methods, the first being usually faster but also more expensive, the second being more sensitive to environmental and operational parameters and cost‐effective. Despite the low pH and potential toxicity of mining waters, heterotrophic denitrification has been successfully applied in the last decade for the treatment of real and simulated mining waters at laboratory scale (Nordström & Herbert, 2017; Zou et al., 2013; Zou et al., 2014). Additionally, some pilot‐scale trials with denitrifying fixed‐bed bioreactors have also been carried out at the mining site (Nordström & Herbert, 2018). However, mining water features concentrations of total organic carbon typically below 10 mg/L (Myagkaya et al., 2016), which may require supplementation of organic matter to enable a complete heterotrophic denitrification, resulting in increased treatment costs and secondary organic pollution. An alternative solution is represented by autotrophic denitrification, applying chemotrophic microorganisms that reduce NO_3_^−^ via oxidation of inorganic electron donors and utilize inorganic carbon for their growth (Di Capua et al., 2019). In recent years, autotrophic denitrification has been also studied at laboratory scale for the treatment of simulated mine waters under low pH and temperature conditions (Di Capua, Lakaniemi, Puhakka, Lens, & Esposito, 2017; Di Capua, Milone, Lakaniemi, Lens, & Esposito, 2017) as well as high concentrations of heavy metals (Di Capua, Milone, Lakaniemi, Hullebusch, et al., 2017), showing outstanding performance with thiosulfate (S_2_O_3_^2−^) as electron donor in fluidized‐bed reactors (FBRs) packed with granular activated carbon (GAC). However, S_2_O_3_^2−^ does not naturally occur in mine waters and its addition would significantly increase the treatment costs. Moreover, S_2_O_3_^2−^ can be rapidly oxidized to sulfate (SO_4_^2−^) by contact with air or disproportionated into elemental sulfur (S^0^) and sulfite (SO_3_^2−^) in acidic media (Druschel & Borda, 2006). S^0^‐packed biocolumns have largely been used for autotrophic denitrification (Asik et al., 2021), but the acidity produced by this process would further reduce the pH and alkalinity of the treated wastewater, leading to fast inhibition of the autotrophic biomass.

Pyrite is a potential electron donor for autotrophic denitrification, and it is typically present in the mine area where it typically responsible for AMD generation (Di Capua et al., 2019). NO_3_^−^ reduction through pyrite oxidation occurs as described by Equation (2) (Torrentó et al., 2010) and may proceed through the biological oxidation of ferrous iron (Fe^2+^) to ferric iron (Fe^3+^) (Equation 3) with subsequent precipitation as ferric hydroxide (Equation 4) (Di Capua et al., 2020).

(2) $$5\;{\mathrm{FeS}}_{2}+14\;{{\mathrm{NO}}_{3}}^{-}+{\text{4H}}^{+}\to 7\;N_{2}+10\;{{\mathrm{SO}}_{4}}^{2-}+5\;{\mathrm{Fe}}^{2+}+{\text{2H}}_{2}O$$

(3) $$2\;{{\mathrm{NO}}_{3}}^{-}+10\;{\mathrm{Fe}}^{2+}+12H^{+}\to N_{2}+10\;{\mathrm{Fe}}^{3+}+6\;H_{2}O$$

(4) $${\mathrm{Fe}}^{3+}+3\;H_{2}O\to \mathrm{Fe}\;{\left(\mathrm{OH}\right)}_{3}+3\;H^{+}$$

Pyrite oxidation to Fe^2+^ consumes 0.8 mol of H^+^ per mol of FeS_2_ (Equation 1), which can increase the pH of the treated water. However, if generation and precipitation of Fe^3+^ occurs, the overall process (Equation 5) yields 1 mol of H^+^ per mol of FeS_2_:

(5) $$30\;{{\mathrm{NO}}_{3}}^{-}+10\;{\mathrm{FeS}}_{2}+20\;H_{2}O\to 15\;N_{2}+20\;{{\mathrm{SO}}_{4}}^{2-}+10\;\mathrm{Fe}\;{\left(\mathrm{OH}\right)}_{3}+10\;H^{+}$$

Previous studies with pyrite‐packed bioreactors showed that the use of pyrite allowed to obtain a stable or even a slightly higher pH in the effluent compared with the influent (Di Capua et al., 2020; Kong et al., 2016; Pu et al., 2015). This is advantageous for the treatment of acidic wastewater, especially if compared with S^0^‐based denitrification which yields 1.06 mol H^+^ per mol of S^0^ (Ucar et al., 2021).

The feasibility of pyrite‐assisted denitrification for AMD treatment could facilitate on‐site treatment, as pyrite is a ubiquitous mineral that can be extracted directly in the mining area. Therefore, in this study, autotrophic denitrification with pyrite was tested at decreasing pH values (from 8 to 3) in a continuous‐flow biofilter to assess process feasibility for nitrogen removal from simulated AMD. The effect of organic carbon presence on the process was also evaluated.

## MATERIALS AND METHODS

### Biofilter operation

A laboratory‐scale recirculated pyrite‐packed biofilter (RPPB) was used to study pyrite‐assisted denitrification of simulated mining water under decreasing pH values. The design, set‐up, and hydrodynamics of the bioreactor as well as microbial enrichment, start‐up phase, and influent composition (except for ethanol concentration) were as described by Di Capua et al. (2020). Prior to this study, the RPPB was inoculated with a culture of *Thiobacillus denitrificans* enriched on S_2_O_3_^2−^ and with activated sludge from a local WWTP and operated for 98 days at 25 (±2) °C. After backwashing, the bioreactor was maintained for 1 month at a hydraulic retention time (HRT) of 8 h and feed pH and alkalinity of around 8 and 700 mg CaCO_3_/L, respectively.

In this study, the RPPB was operated under nine different operational periods at different HRTs and feed organic carbon and pH conditions (Table 1). During period I (days 0–16), the bioreactor was fed with organic‐free synthetic influent and maintained at an HRT of 8 h. After period I, the influent was supplemented with 10 mg/L of pure (>99.8%) ethanol (Sigma‐Aldrich, USA), corresponding to a dissolved organic carbon (DOC) concentration of around 5.2 mg/L, and the bioreactor performance was evaluated at HRTs of 8 h (period II) and 5 h (period III). During period IV, ethanol concentration in the feed was increased to 20 mg/L, which was maintained until the end of the study. From period V to period IX, the influent pH was gradually decreased from around 8 to 3 by adding 37% HCl, and pyrite‐assisted denitrification was studied under acidic conditions at a stable HRT of 8 h. The RPPB and influent tanks were periodically sampled to measure the concentrations of NO_3_^−^, nitrite (NO_2_^−^), SO_4_^2−^, S_2_O_3_^2−^, sulfide, DOC, and alkalinity. Biofilm‐coated pyrite granules were collected from the RPPB at the end of the study from different bed heights to evaluate biofilm development and distribution within the biofilter.

**TABLE 1:** Experimental conditions applied to the RPPB during the study

Period	Time (days)	HRT (h)	Ethanol (mg/L)	pH	Alkalinity (mg CaCO_3_/L)	Nitrogen loading rate (g N–NO_3_^−^/m^3^ h)
I	0–16	8	0	7.7 (±0.1)	663 (±15)	2.0 (±0.1)
II	17–31	8	10	7.9 (±0.2)	692 (±19)	1.9 (±0.2)
III	32–49	5	10	8.0 (±0.2)	698 (±7)	3.0 (±0.3)
IV	50–64	5	20	7.9 (±0.2)	688 (±12)	3.2 (±0.1)
V	65–73	8	20	7.9 (±0.1)	690 (±9)	1.9 (±0.1)
VI	74–91	8	20	6.7 (±0.1)	348 (±31)	2.0 (±0.2)
VII	92–117	8	20	5.7 (±0.1)	69 (±22)	2.0 (±0.2)
VIII	118–142	8	20	4.7 (±0.2)	10 (±5)	1.9 (±0.1)
IX	143–148	8	20	3.0 (±0.1)	0	1.9 (±0.1)

### Analytical methods

The concentrations of NO_3_^−^, NO_2_^−^, SO_4_^2−^ and S_2_O_3_^2−^, dissolved oxygen (DO), alkalinity, and sulfide (free and acid‐volatile) as well as pH were measured as described by Di Capua et al. (2020). DOC concentration was analyzed as described by Iannacone et al. (2021). DO concentration and pH were monitored directly in the bioreactor and influent tanks. Alkalinity and sulfide levels were monitored in unfiltered liquid samples, whereas the concentrations of oxyanions and DOC were measured after sample filtration with Minisart 0.45 μm syringe filters (Sartorius, Germany). Biofilm quantification was performed by persulfate digestion and subsequent evaluation of total nitrogen (TN) content. Pyrite granules were collected from the bioreactors at the end of the study and washed with ultrapure water. Equal volumes of pyrite and ultrapure water were added to falcon tubes and the biomass was detached by 5‐min sonication in an ultrasonic bath (VWR, USA) followed by manual shaking. The detached solids were pelletized by centrifugation at 4500 rpm for 10 min. The pellet was added to a digestion tube with 4 ml of ultrapure water and 2 ml of an alkaline potassium persulfate (K_2_S_2_O_8_) solution (0.15 M). After digestion and cooling, the tube content was centrifuged, and the supernatant analyzed via ion chromatography to evaluate TN as NO_3_^−^. Finally, biomass concentration was evaluated based on the obtained TN according to the formula C_5_H_7_O_2_N.

### Statistical analysis

A one‐way analysis of variance (ANOVA) was performed using the Data Analysis Tool of Excel 2016 (Microsoft, USA) to determine the statistical differences in effluent NO_3_^−^ concentration of each operational period. The significant difference was considered at 95% (*p* < 0.05).

## RESULTS AND DISCUSSION

### Impact of organic supplementation and HRT on pyrite‐assisted denitrification

**FIGURE 1:**
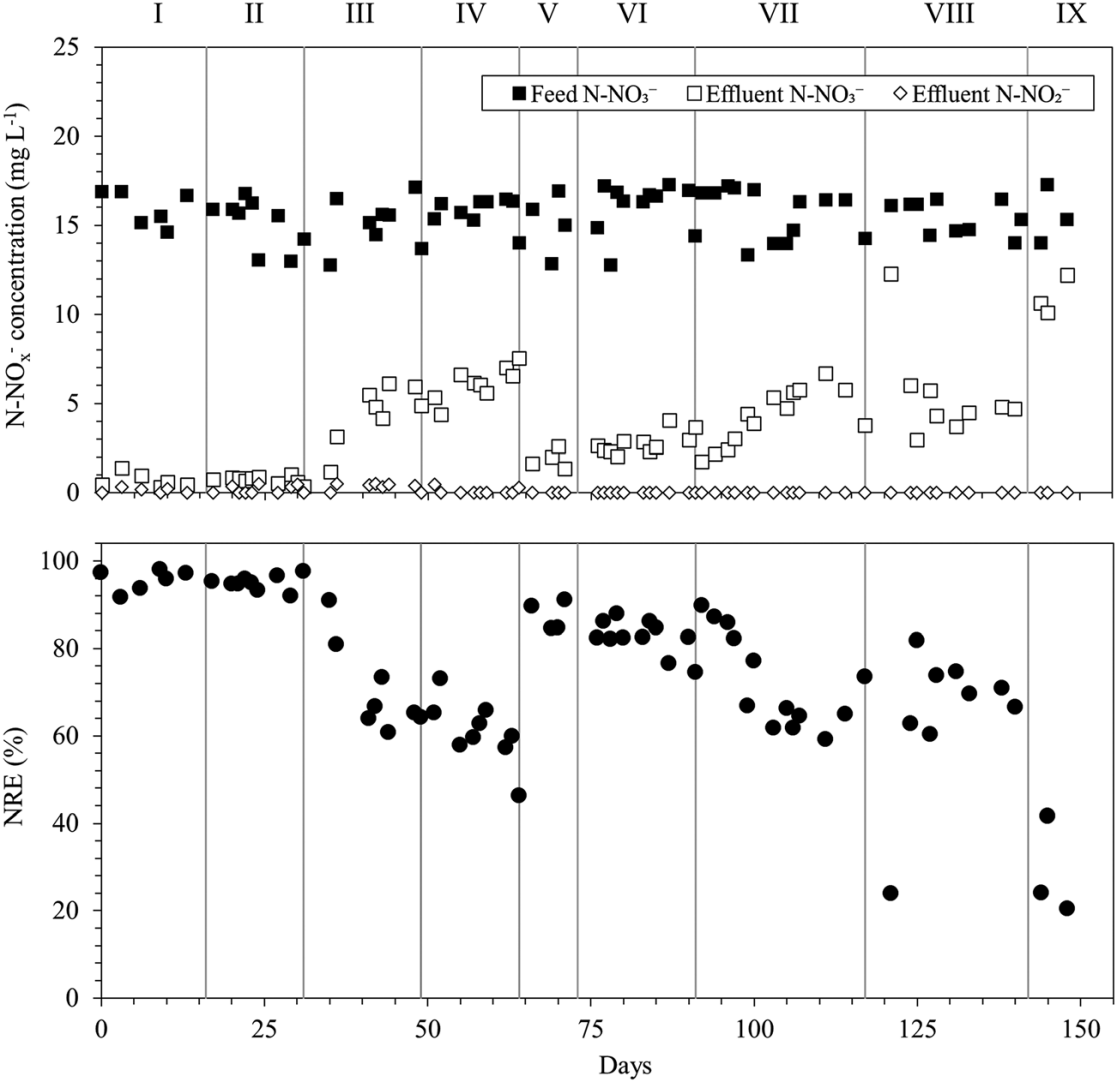
Profiles of NO_3_^−^ and NO_2_^−^ concentrations and NRE in the RPPB during the study

**FIGURE 2:**
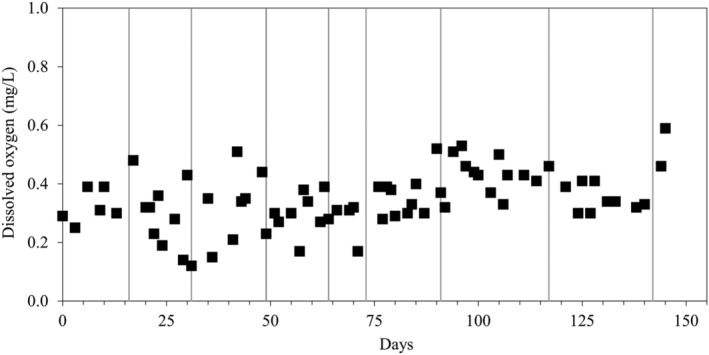
DO profile in the RPPB during the study

During periods I–IV, the RPPB was operated at a stable feed pH of around 8 (Table 1) to evaluate the effect of organic carbon addition and HRT decrease on the denitrification process. The concentration profiles of nitrogen oxyanions (NO_3_^−^ and NO_2_^−^) and the nitrogen removal efficiency (NRE) of the RPPB are represented in Figure 1. During the first 2 periods, the HRT, feed alkalinity concentration, and nitrogen loading rate (NLR) in the RPPB were maintained stable at around 8 h, 700 mg CaCO_3_/L and 2 g N–NO_3_^−^/m^3^ h (Table 1), whereas ethanol concentration in the feed was increased from 0 mg/L (period I) to 10 mg/L (period II). DO levels in the RPPB remained below 0.6 mg/L throughout the entire study assuring anoxic conditions (Figure 2). During the first 2 periods, the effluent NO_3_^−^ in the RPPB remained stable at concentrations ≤1 mg N–NO_3_^−^/L (*p* > 0.05), resulting in NREs >90% (95%–96% on average) (Figure 1). The visual observation of gas bubbles entrapped in the pyrite bed and eventually released to the atmosphere suggested that N_2_ was generated during the process. However, no N_2_ nor nitrous oxide (N_2_O), a potential and hazardous denitrification intermediate (Sabba et al., 2018), were quantified in this study. The addition of 10 mg/L of ethanol did not show a significant effect on the denitrification performance of the RPPB. In contrast, the impact of HRT decrease from 8 h (period II) to 5 h (period III) was evident (*p* < 0.05), as N–NO_3_^−^ levels in the effluent progressively increased up to 5.6 mg/L, reducing the NRE to around 60% by the end of period III. This behavior is similar to that observed by Di Capua et al. (2020) in a RPPB operated under completely autotrophic conditions when the HRT was decreased from 5 to 2 h and the NLR increased from 3.0 to 7.7 g N–NO_3_^−^/m^3^ h. In the present study, a lower NLR (1.9 g N–NO_3_^−^/m^3^ h, Table 1) could be sustained by the RPPB while maintaining a high NRE (>90%), which might be due to a lower amount of autotrophic biomass in the biofilter. Biofilm quantification performed at the end of the study showed that 1.8 (±0.3) g cell/L pyrite covered the pyrite granules along the biofilter. However, biomass concentration was not evaluated in the previous study (Di Capua et al., 2020), and therefore, a comparison is not possible. Li et al. (2021) measured a biofilm concentration of 8.05 g VSS/L pyrite in an upflow pyrite biofilter, which was not far from the concentration measured in this study.

Increase of ethanol concentration in the feed from 10 mg/L (period III) to 20 mg/L (period IV) did not enhance NRE, which remained at an average value of 61 (±7) %. This result indicates that supplementing 20 mg/L of ethanol was not sufficient to boost heterotrophic nitrate removal in the biofilter. This could be due to the long‐term cultivation of the RPPB biofilm under complete autotrophic conditions during previous operation, which could select for specific autotrophic biomass. Moreover, natural pyrite features a low degree of biomass colonization, which results in slow and limited biomass development (Torrentó et al., 2012). Although *T. denitrificans* could establish active colonies on the pyrite surface, heterotrophic bacteria were likely washed out from the biofilter during previous reactor operation. Increasing the HRT from 5 h (period IV) to 8 h (period V) rapidly recovered the denitrification efficiency, which reached values ≥85%. Nevertheless, by comparing the NREs obtained at periods II and V, it is evident that previous operation at lower HRT (and higher NLR) had negatively impacted the performance of the bioreactor. This could be linked to the higher shear stress caused by the increase of flow rate applied to reduce the HRT, which may have detached some biofilm and decreased the amount of active biomass in the system.

### Effect of acidic pH on pyrite‐based denitrification in the RPPB

**FIGURE 3:**
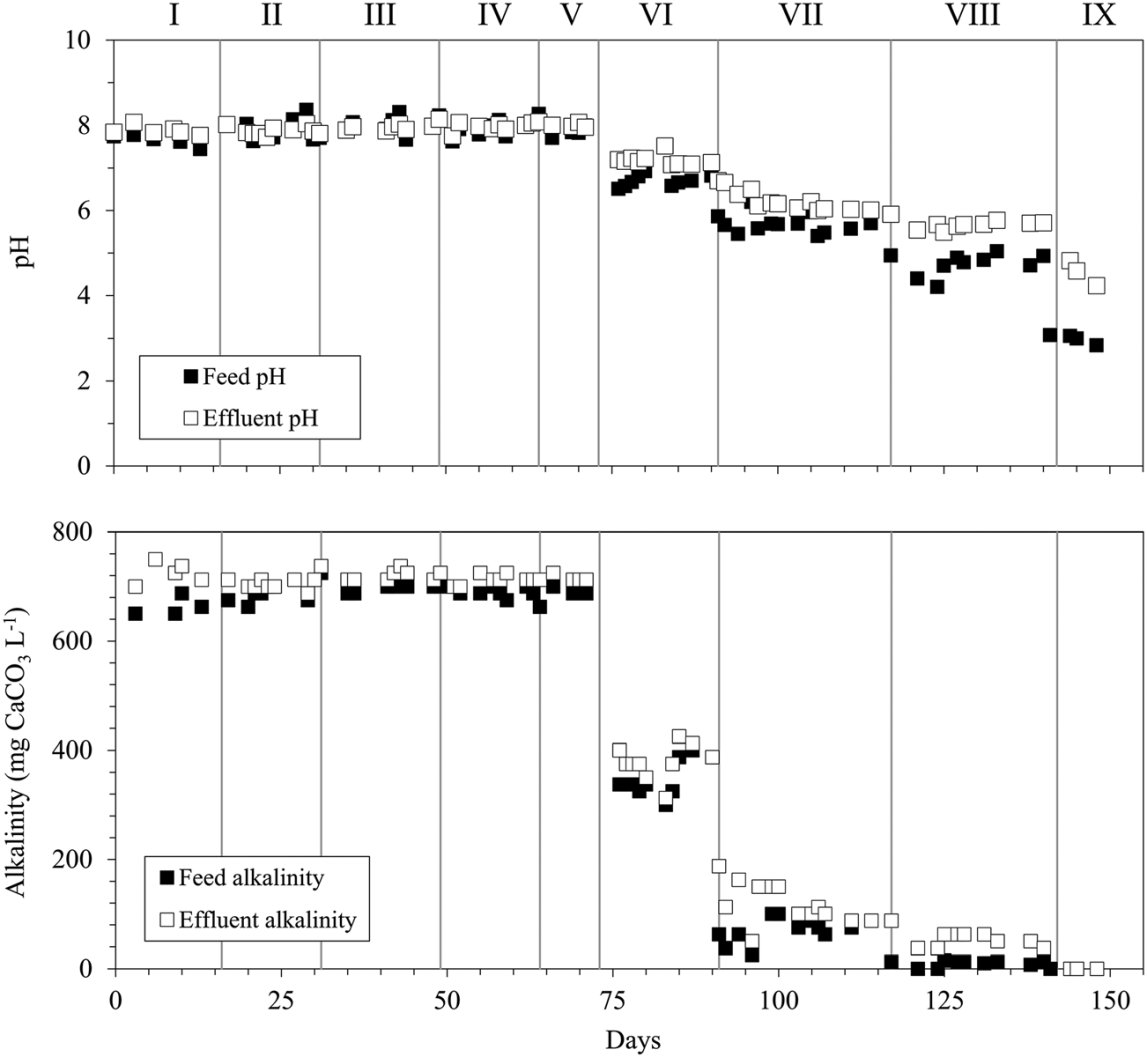
pH and alkalinity profiles of the RPPB influent and effluent during the study

From period V to period IX, the effect of acidic pH on NO_3_^−^ removal was evaluated at decreasing influent pH values while maintaining the NLR at around 2 g N–NO_3_^−^/m^3^ h (HRT = 8 h) (Table 1). The trends of pH and alkalinity in the RPPB and synthetic wastewater are shown in Figure 3. In period V, no acid was added to the influent and the pH was maintained at the initial value of 7.9 (±0.1), resulting in NRE ≥ 85% (Figure 1). Decrease of feed pH to 6.7 (±0.1) in period VI only slightly reduced the NRE, which remained above 80% with an average value of 83 (±3) %. When the feed pH was lowered to 5.7 (±0.1) in period VII, the NRE progressively decreased to around 60% (days 103–111) prior to increase to 73% at the end of the period. These results are in line with those reported by Liu and Koenig (2002) in batch experiments with S^0^, reporting progressive inhibition of autotrophic denitrification at pH below 6.8 and complete inhibition at pH 6. In another study, Di Capua, Lakaniemi, Puhakka, Lens, and Esposito (2017) reported that almost complete denitrification was possible even at pH below 6 in a FBR packed with GAC and fed with S_2_O_3_^2−^, which was attributed to a gradual microbial adaptation to low pH and to the establishment of pH gradients within the thick GAC‐attached biofilm. These mechanisms unlikely occurred in the RPPB, as pH decrease in the feed (from 7.9 to 5.7 in 19 days, Table 1) was faster and more pronounced than in the FBR (from 7.0 to 5.75 in 63 days), and biofilm thickness in the RPPB was certainly lower due to the much lower specific surface area of natural pyrite granules (<1 m^2^/g) compared to GAC (>1000 m^2^/g).

Further decrease of feed pH to 4.7 (±0.2) in period VIII caused a breakthrough of N–NO_3_^−^ concentration up to 12.3 mg/L (Figure 1). However, NO_3_^−^ levels immediately returned to values similar to those observed at the end of the previous period, indicating that the performance of the bioreactor was not irreparably worsened at pH 4.7. In contrast, the denitrification efficiency was strongly worsened when the pH of the influent was finally lowered to 3.0 (period IX), as NRE decreased to 20%–42%. It should be highlighted that no alkalinity was present in the influent medium at a pH as low as 3.0 (Table 1, Figure 3), as all inorganic carbon fed with the influent as NaHCO_3_ is converted to H_2_CO_3_/CO_2 (aq)_ at pH ≤ 4.5. Indeed, extremely low alkalinity, i.e., 10 (±5) mg CaCO_3_/L in the feed and 52 (±11) mg CaCO_3_/L in the effluent, was measured already at feed pH of 4.7 (Figure 3), although it seemed sufficient to sustain microbial activity at acceptable NREs (>60%). Besides the NaHCO_3_ supplemented with the feed, the soluble CO_2_ in the influent generated by acid addition may have also served as carbon source for autotrophic bacteria at low pH values and could sustain little denitrification activity at a feed pH as low as 3.0.

The effluent pH in periods I–V remained stable at 7.9 (±0.1) (similar to the feed pH), whereas effluent alkalinity (715 ± 13 mg CaCO_3_/L) was slightly higher than in the feed (689 ± 17 mg CaCO_3_/L), confirming the proton‐buffering action of pyrite‐assisted denitrification indicated by Equation (1). During periods VI–IX, the effluent pH was 0.5–1.5 units higher than the feed pH. This difference can be attributed also to the increased amount of acid added to lower the pH, leading to a larger amount of CO_2_ produced in the influent tank and subsequently stripped in the RPPB. As a result of pyrite buffering and CO_2_ stripping, the effluent pH remained always >4 even when the pH in the feed was lowered to 3.0 (period IX). In period VIII (feed pH 4.7), the effluent pH and alkalinity concentration remained ≥5.5 and 50 mg CaCO_3_/L, respectively, which may explain the relatively high NRE (>60%) observed during this period.

### Evolution of sulfur species and DOC in the RPPB

**FIGURE 4:**
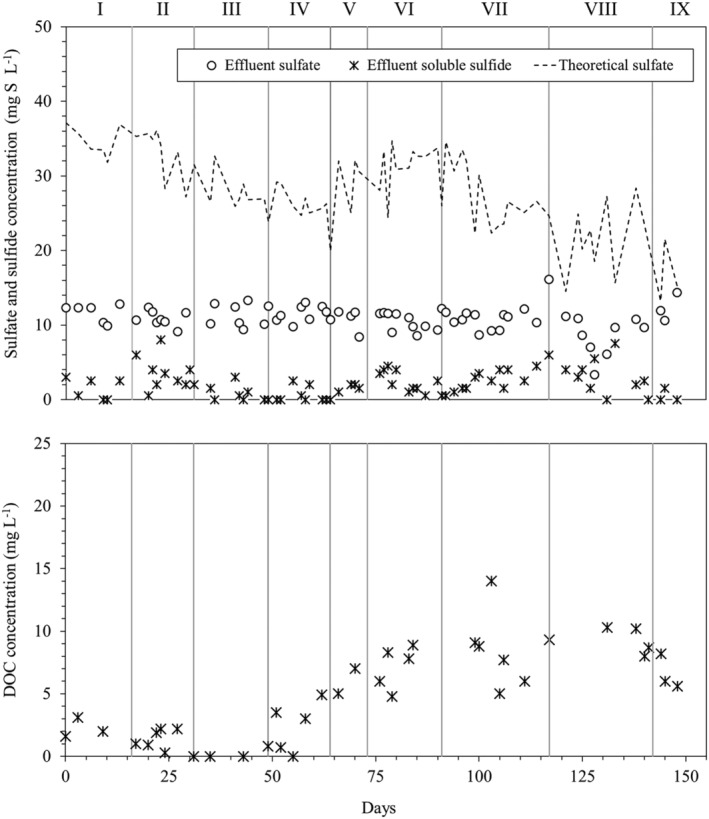
Temporal profiles of S–SO_4_^2−^, soluble S^2−^, and DOC concentrations in the RPPB. The dashed line represents the theoretical profile of the effluent S–SO_4_^2−^ concentration

Figure 4 shows the concentration profiles of the monitored sulfur species and DOC in the bioreactor during the study. Effluent sulfate level in period I was 12 (±1) mg S–SO_4_^2−^ L^−1^, which was much lower than the theoretical concentrations calculated according to Equation (1) and only slightly higher than influent levels (11 ± 1 mg S–SO_4_^2−^ L^−1^). Apparently, little SO_4_^2−^ was produced during the denitrification process, likely due to the incomplete oxidation of FeS_2_ to S^0^ or SO_3_^2−^ (Zhang et al., 2012). As reported by Druschel and Borda (2006), the presence of significant amount of S^0^ as a result of pyrite oxidation has been noted in several studies. Alternatively, the produced SO_4_^2−^ could be partly reduced to sulfide, or intermediates (e.g., S_2_O_3_^2−^ or SO_3_^2−^) by sulfate‐reducing bacteria (SRB) (Sabba et al., 2016; Tong et al., 2017) and/or precipitated with iron (Di Capua et al., 2020). However, it should be noted that no S_2_O_3_^2−^ was detected in the RPPB effluent during the study. Ethanol supplementation from period II on had no significant impact on effluent SO_4_^2−^ levels, which suggests that biological SO_4_^2−^ reduction was not the main mechanism for the low SO_4_^2−^ levels in the effluent, as SRB typically utilize organic carbon. Similarly, increase of acidity in the system during periods V–IX did not change SO_4_^2−^ levels significantly, except for period XIII when a drop in effluent SO_4_^2−^ concentration was observed. However, effluent SO_4_^2−^ levels rose to initial levels during the last period, when feed pH was the lowest tested and NRE was <50%. This further suggests that pyrite was mainly oxidized to intermediate sulfur compounds such as S^0^ and SO_3_^2−^ rather than SO_4_^2−^. However, this cannot be confirmed as neither S^0^ nor SO_3_^2−^ were monitored in the RPPB during this study.

Sulfide was detected in the RPPB at concentrations up to 8 mg/L (Figure 4). Sulfide generation could occur due to SRB activity and/or S^0^/S_2_O_3_^2−^ disproportionation. When 20 mg/L of ethanol was supplied to the feed in period IV, sulfide levels in the effluent were quite low (<3 mg/L), suggesting that organic addition did not stimulate SRB activity. Therefore, it is probable that sulfide was produced through disproportionation of S^0^ and/or S_2_O_3_^2−^ generated by the partial oxidation of the pyrite mineral or indirect attack mechanism (Schippers et al., 1996). Similar to DOC, increase of acidity in the system did not significantly affect sulfide levels in the bioreactor. Indeed, the lowest levels of sulfide in the entire study were observed in period IX (≤1.5 mg/L), when autotrophic denitrification was severely inhibited, indicating that sulfide production was linked to pyrite oxidation.

DOC levels in the effluent remained <4 mg/L during the first three experimental periods (Figure 4). Increase of feed ethanol concentration from 10 to 20 mg/L at the beginning of period IV led to an increase of effluent DOC levels to around 5 mg/L by the end of the period. Increase of acidity during periods V–IX further increased the levels of DOC in the bioreactor, which reached a maximum value of 14 mg/L in period VII. DOC increase was likely promoted by the release of soluble microbial products from the biofilm in response to the increase of acid supplementation to the feed, leading to death and detachment of the cells covering the outer layer of the biofilm. Indeed, biofilm systems allow the protection of the internal layers of bacteria and the establishment of pH gradients within the biofilm, providing a greater resistance to harsh conditions compared with suspended‐growth systems (Di Capua, Lakaniemi, Puhakka, Lens, & Esposito, 2017).

## CONCLUSIONS

Pyrite‐assisted denitrification in a RPPB is a valid strategy for the remediation of acidic nitrate‐contaminated waters. However, satisfactory results could be achieved at HRT > 5 h and feed pH > 3, resulting in NRE > 60%. The RPPB performance was relatively stable at feed pH values from 8 to 4.7, whereas it severely deteriorated at a feed pH of 3.0. Addition of organic carbon showed no significant effects on reactor performance nor supported heterotrophic bioprocesses significantly, although it slightly increased DOC concentration in the effluent. Based on these results, the RPPB could be applied for the remediation of mildly acidic wastewaters.

### AUTHOR CONTRIBUTIONS

**Francesco Di Capua:** Conceptualization; data curation; formal analysis; investigation; methodology; visualization. **Giovanni Esposito:** Funding acquisition; project administration; resources; supervision.

## Data Availability

The data that support the findings of this study are available on request from the corresponding author. The data are not publicly available due to privacy or ethical restrictions.
